# Analysis of Brain Lesion Impact on Balance and Gait Following Stroke

**DOI:** 10.3389/fnhum.2019.00149

**Published:** 2019-05-14

**Authors:** Shirley Handelzalts, Itshak Melzer, Nachum Soroker

**Affiliations:** ^1^Department of Physical Therapy, Faculty of Health Sciences, Ben-Gurion University of the Negev, Beer-Sheva, Israel; ^2^Loewenstein Rehabilitation Hospital, Ra’anana, Israel; ^3^Sackler Faculty of Medicine, Tel-Aviv University, Tel-Aviv, Israel

**Keywords:** reactive balance control, voxel-based lesion-symptom mapping, falls, fall threshold, perturbations, rehabilitation

## Abstract

Falls are a leading cause of serious injury and restricted participation among persons with stroke (PwS). Reactive balance control is essential for fall prevention, however, only a few studies have explored the effects of lesion characteristics (location and extent) on balance control in PwS. We aimed to assess the impact of lesion characteristics on reactive and anticipatory balance capacity, gait, and hemiparetic lower limb function, in PwS. Forty-six subacute PwS were exposed to forward, backward, right and left unannounced horizontal surface translations in six increasing intensities while standing. Fall threshold (i.e., perturbation intensity that results in a fall into the harness system) was measured. In addition, the Berg Balance Scale (BBS), 6 Minute Walk Test (6MWT) and Lower Extremity Fugl-Meyer (LEFM) were measured. Lesion effects were analyzed separately for left and right hemisphere damaged (LHD, RHD) patients, using voxel-based lesion-symptom mapping (VLSM). Our results show that voxel clusters where damage exerted a significant impact on balance, gait and lower-limb function were found in the corticospinal tract (CST), in its passage in the corona radiata and in the posterior limb of the internal capsule. An additional significant impact was found to lesions affecting the putamen and the external capsule (EC). Balance, gait, and hemiparetic lower limb function showed much overlap of the corresponding “significant” voxel clusters. Test scores of RHD and LHD patients were affected largely by damage to homologous regions, with the LHD group showing a wider distribution of “significant” voxels. The study corroborates and extends previous findings by demonstrating that balance control, gait, and lower limb function are all affected mainly by damage to essentially the same brain structures, namely—the CST and adjacent structures in the capsular-putaminal region.

## Introduction

Falls occur in up to 70% of stroke victims during the first 6 months after discharge from hospital or rehabilitation setting (Forster and Young, 1995; Davenport et al., 1996; Weerdesteyn et al., 2008; Batchelor et al., 2012). Compared with a general population of older adults who fall, persons with stroke (PwS) who fall are twice as likely to sustain a hip fracture (Forster and Young, 1995; Langhorne et al., 2000; Pouwels et al., 2009; Winstein et al., 2016). In addition to physical consequences associated with fractures and related injuries, falls may have serious psychological and social consequences such as functional decline, poor quality of life, dependency, social isolation and depression (Winstein et al., 2016).

Balance control is a strong predictor of functional recovery, walking capacity and fall risk after stroke (Michael et al., 2005; Belgen et al., 2006; Simpson et al., 2011; van Duijnhoven et al., 2016; Xu et al., 2018). Commonly used clinical measures [e.g., the Berg Balance Scale (BBS), Dynamic Gait Index (DGI) and Timed Up and Go (TUG)] focus on anticipatory balance control, that is essential for the maintenance of postural stability prior to voluntary movement by compensating for destabilizing forces associated with the movement. However, in situations of unexpected loss of balance, the ability to respond effectively (i.e., reactive balance control), is crucial for fall prevention (Maki and McIlroy, 1997). After small external disturbances, we can usually regain balance while keeping the feet in place. However, falls often occur from large external disturbances (Maki and McIlroy, 2006) which require a rapid step response to alter the base of support. Recent studies assessed reactive balance control abilities by exposure to external perturbations delivered from a movable platform (Salot et al., 2015; Honeycutt et al., 2016; de Kam et al., 2017). In this paradigm, the time, direction and intensity of perturbation is unpredicted, thus simulating situations in real life where loss of balance is unexpected. PwS have shown substantially impaired reactive balance responses compared to healthy individuals, characterized by increased need for external assistance, difficulty initiating protective stepping with either lower limb, increased usage of multiple step strategy, and more falls into the harness system (Marigold and Eng, 2006; Mansfield et al., 2013; Martinez et al., 2013; Inness et al., 2014; Salot et al., 2015; Honeycutt et al., 2016; de Kam et al., 2017).

Although impairments in balance control following stroke have been studied extensively and their impact on the risk of falls and fractures has been established, relatively few studies have explored the associations between these impairments and damage to specific brain structures. Voxel-based lesion symptom mapping (VLSM) is a commonly used method for analyses of the neural basis underlying different types of impairment described by Bates et al. (2003). Use of VLSM for analysis of lesion characteristics in lower-limb paresis, gait instability and impaired balance, is much less prevalent compared with its use in analyses focusing on the hemiparetic upper limb. Using VLSM, Reynolds et al. (2014) found that lower BBS scores were associated with damage in the precentral gyrus, putamen, caudate and pallidum, cuneus, frontal operculum, and also damage to some thalamic structures. They also found that TUG scores were associated with lesions in the postcentral gyrus, insular cortex, superior temporal cortex, and the inferior parietal lobule. Lee et al. (2017) found that lesions involving the corona radiata, internal capsule, globus pallidus, putamen, primary motor cortex and caudate nucleus are associated with poor recovery of gait, as measured with the functional ambulation category (FAC) 6 months after stroke onset. In contradiction to the above findings, Moon et al. (2016) found no specific lesion locations in association with poor BBS and FAC scores. Poor gait speed was found to associate with damage to the putamen, insula, caudate, corona radiata and external capsule (EC; Reynolds et al., 2014; Jones et al., 2016). Alexander et al. (2009) found that damage to the putamen, insula and EC was related to gait asymmetry in chronic PwS. Lower Extremity Fugl-Meyer (LEFM) scores were found to be associated with damage in the corona radiata, putamen, globus pallidus, caudate, insula and internal capsule (Reynolds et al., 2014; Moon et al., 2016).

Lesion studies investigating the effects of stroke location on motor ability often address the right and left hemispheres as two parallel and analogous systems, and flip lesions onto a single hemisphere template (Lo et al., 2010; Zhu et al., 2010; Cheng et al., 2014; Meyer et al., 2015). This practice may obscure important differences between the hemispheres. A recent VLSM study showed that LEFM scores are affected by a wider lesion distribution in the left hemisphere compared to the right hemisphere (Moon et al., 2016). In contrast, a *post hoc* VLSM analysis aimed to assess hemispheric effects (Jones et al., 2016), revealed no “significant” voxel clusters in either hemisphere. Considering the relative paucity of studies addressing lesion effects on balance control and gait and the fact that most of the existing studies did not analyze right and left hemisphere damage separately, our objective in the current study was to explore the impact of lesion location, in each hemisphere, on reactive and anticipatory balance capacity, gait, and hemiparetic lower limb function, in PwS.

## Materials and Methods

### Participants

Forty-six first-event subacute stroke patients (<3 months after onset) were recruited for the study during their hospitalization at the Loewenstein Rehabilitation Hospital (LRH), Ra’anana, Israel, as part of a research project aimed to characterize reactive balance responses in PwS. PwS were included if they were able to stand for at least 2 min and to walk independently or under supervision with or without a walking aid. Exclusion criteria included unstable clinical/metabolic state, existence of other neurological disorders in addition to stroke, significant musculoskeletal conditions (e.g., severe arthritis, joint replacement surgery), significant visual impairment, inability to follow verbal instructions due to aphasia, and existence of significant unilateral spatial neglect in clinical testing. Patients were also excluded if their CT scan revealed brain pathology other than the recent stroke (e.g., existence of old lacunar infarctions, diffuse white matter changes, brain atrophy, etc). The study was approved by the LRH Review Board (#LOE-14-0021). All participants were informed about the protocol and gave their written informed consent prior to inclusion in the study.

### Clinical Assessment

**Figure 1:**
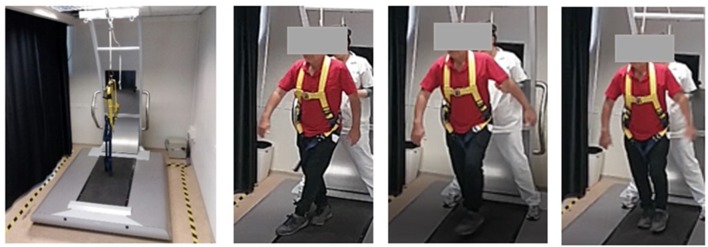
Setup for reactive balance control assessment. On the left is the computerized treadmill system. On the right is a video sequence demonstrating reactive step response to lateral surface translation of a stroke patient with right hemiparesis. A written informed consent was obtained from the individual for the publication of this image.

To assess reactive balance capacity after simulated loss of balance, participants stood on a computerized treadmill system with a horizontal movable platform (Balance Tutor, *MediTouch*, Israel), wearing a safety harness that prevented falls but did not restrict their movements (Figure 1). They were instructed to stand with feet placed together and to react naturally to prevent themselves from falling in response to random unannounced forward, backward, right and left surface translations. Surface translation intensity was increased systematically in a graded manner from low (intensity 1) to high (intensity 6) for a total of 24 perturbation trials (characteristics of perturbation intensities are described in Table 1). In case of insufficient balance response i.e., a fall into harness, participants did not continue to a higher intensity. We measured the fall threshold, defined as the perturbation intensity that results in unsuccessful balance recovery, i.e., when a subject is unambiguously supported by the harness system (Honeycutt et al., 2016).

**Table 1:** Perturbation parameters by perturbation intensity and direction.

Perturbation Intensity	Forward/Backward surface translation				Rightward/Leftward surface translation			
	Distance (cm)	Velocity (cm/s)	Acceleration (cm/s^2^)	Duration (s)	Distance (cm)	Velocity (cm/s)	Acceleration (cm/s^2^)	Duration (s)
1	10.44	17.62	126.89	0.41	5.68	20.17	73.27	0.39
2	14.17	24.93	150.34	0.43	8.03	27.75	97.41	0.41
3	17.89	32.24	173.79	0.45	10.37	35.34	121.55	0.41
4	21.62	39.55	197.24	0.47	12.72	42.93	145.68	0.42
5	25.34	46.86	220.69	0.48	15.06	50.51	169.82	0.42
6	29.06	54.17	244.13	0.49	17.41	58.10	193.96	0.42

*The system controller receives from the PC program the required motion parameters, which are the target position, maximal velocity, acceleration and deceleration. The controller has an internal motion profile generator that generates a trapezoidal velocity profile. Abbreviation: cm, centimetre; cm/s, centimetre per second; cm/s^2^, centimetre per second square. Presented are peak values*.

To assess motor impairments, functional balance and gait abilities we used the LEFM scale, BBS and 6 Minute Walk Test (6MWT). The LEFM is a set of tasks pertaining to a range of motion, muscle strength, and reflexes of the affected lower limb. The maximum score is 34; lower scores signify motor impairment (Fugl-Meyer et al., 1975). The BBS consists of 14 tasks (e.g., reaching, turning and balancing on one leg), designed to assess static balance and fall risk in adult populations. The maximum score is 56 indicating better balance (Berg et al., 1995). Gait function was assessed using the 6MWT that measures the distance in meters that subjects can walk in 6 min (Butland et al., 1982). Those measures have been found to demonstrate strong inter-rater and test–retest reliability in PwS (Duncan et al., 1992; Berg et al., 1995; Eng et al., 2004; Flansbjer et al., 2005, 2012). Participants performed all tests wearing their own sport shoes and foot orthosis in case they needed ankle support.

### Lesion Analysis

CT scans dated on average 22 days post stroke onset were examined by a physician experienced in the analysis of neuro-imaging data (NS). This was done in order to ensure that lesion boundaries were clear and traceable and that the CT presents a stable pattern of tissue damage without a mass effect from residual edema. Lesion analyses were performed with the Analysis of Brain Lesions (ABLe) module implemented in MEDx software (Medical-Numerics, Sterling, VA, USA). Lesion delineation was done manually on the digitized CTs. ABLe characterizes brain lesions in CT scans of adult human brain by spatially normalizing the lesioned brain into Talairach space using the Montreal Neurological Institute (MNI) template. It reports tissue damage in the normalized brain using an interface to the Talairach Daemon (San Antonio, Texas), Automated Anatomical Labeling (AAL) atlas, Volume Occupancy Talairach Labels (VOTL) atlas or the White Matter Atlas (Lancaster et al., 2000; Tzourio-Mazoyer et al., 2002; Solomon et al., 2007). Quantification of the amount of tissue damage within each structure/region of the atlas was obtained as described by us earlier (Haramati et al., 2008). Registration accuracy of the scans to the MNI template across all subjects was 94.1% (94.28 ± 1.02 and 93.8 ± 1.4 in RHD and LHD patients, respectively).

### Voxel-based Lesion-Symptom Mapping (VLSM)

VLSM (Bates et al., 2003) was used to identify voxels (1 × 1 × 1 mm) of the normalized brain where damage exerted a significant effect on balance, gait, and lower extremity motor function. We computed a map of *z* values, where the value of each voxel represents the z-score that compares subjects’ clinical measures with lesions to those without lesions, and identified a peak z-value within each significant cluster. Comparisons were made using z-scores derived from *t*-test for normally distributed variable (i.e., 6MWT) and Mann-Whitney U test for ordinal or non-normally distributed variables (i.e., fall threshold, BBS and LEFM). In order to avoid spurious results due to low numbers of lesioned voxels, only voxels lesioned in at least 10 participants were tested (Rorden et al., 2007; Medina et al., 2010) and at least 10 adjacent voxels had to show a statistically significant impact on performance for a cluster to be reported (McDonald et al., 2017). A voxel-wise false discovery rate (FDR; Benjamini and Hochberg, 1995) correction for multiple comparisons was controlled to be less than 0.05. Since there may be multiple voxels with this maximum z-score in the cluster, we report the coordinate of the voxel that is most superior, posterior and left in its location within the cluster (the centroid of the cluster is not reported as it may not have the highest z-score value and it may not be an above-threshold voxel). The AAL atlas for gray matter and the white Matter Atlas (Lancaster et al., 2000; Tzourio-Mazoyer et al., 2002; Solomon et al., 2007) were used to identify the location of the significant clusters.

### Statistical Analysis of Clinical Data

Statistical analyses were performed using IBM SPSS version 24.0 (IBM Corp, NY, USA). The Shapiro-Wilk Test was used to test the assumption of normal distribution (*p* > 0.05). Baseline characteristics for behavioral data were compared using independent-samples *t*-test for continuous variables, Mann-Whitney U test for ordinal variables or variables with non-normal distributions and Chi-square test for categorical data. Correction for multiple comparisons was conducted using the Bonferroni correction (*p* = 0.05/12 = 0.004).

## Results

Patients’ demographics and clinical scores are presented in Table 2. No significant differences were found in clinical and demographic parameters of individuals with left and right hemisphere damage.

**Table 2:** Demographic and clinical characteristics of participants.

	RHD group (*n* = 24)	LHD group (*n* = 22)	*p*-value
Gender (M/F)	18/6	18/4	0.575^c^
Age (years)	61.5 (8.3)	60.9 ± 8.9	0.815^a^
Weight (kg)	77.3 (16.2)	74.2 ± 11.5	0.451^a^
Height (cm)	170.2 (8.6)	168.9 ± 9.8	0.613^a^
Lesion type (I/H)	20/4	17/5	0.605^c^
Lesion volume (cc)	10.1 (14.4)	12.7 (13.4)	0.159^b^
TAO to clinical assessment (days)	43.0 (19.4)	49.9 (15.7)	0.159^b^
Use of assistive device for walking (No/Yes)	3/21	4/18	0.592^c^
LEFM (0–34)	30.5 (4.6)	28.3 (4.1)	0.036^b^
BBS (0–56)	43.6 (10.9)	42.4 (8.1)	0.338^b^
6MWT (meters)	297.3 (136.4)	280.1 (145.9)	0.690^a^
Fall threshold (perturbation intensity)	4.2 (2.1)	4.3 (2.4)	0.832^b^

*Mean ± (SD) values. Abbreviations: RHD, right hemisphere damage; LHD, left hemisphere damage; Gender, M-male, F-female; Lesion type, I-ischemic, H-hemorrhagic; TAO, time after onset; LEFM, Lower Extremity Fugl-Meyer Scale; BBS, Berg Balance Scale; 6MWT, 6 Minute Walk Test. ^a^t-test, ^b^Mann-Whitney U, ^c^Chi-square. Bonferroni correction for multiple comparisons was applied to adjust the significance level, *p* < 0.004*.

**Figure 2:**
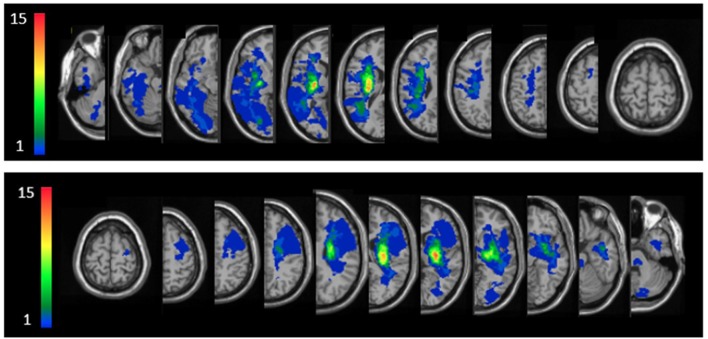
Lesion overlay maps of right hemisphere damaged (RHD, *n* = 24) and left hemisphere damaged (LHD, *n* = 22) groups—top and bottom rows, respectively. Representative normalized slices (out of 90 normalized slices employed) are displayed in radiological convention (right hemisphere on left side and vice versa), with warmer colors indicating greater lesion overlap (units: number of patients with lesion in the colored region).

Individual lesion data are displayed in Supplementary Figure S1. Overlay lesion maps (stroke lesion distribution) of left hemisphere damaged (LHD) and right hemisphere damaged (RHD) patients are presented in Figure 2. As can be seen, in both groups the maximal lesion overlap was in the capsular-putaminal region. Comparisons between groups for the locations of lesions are presented in Supplementary Tables S1A,B.

**Figure 3:**
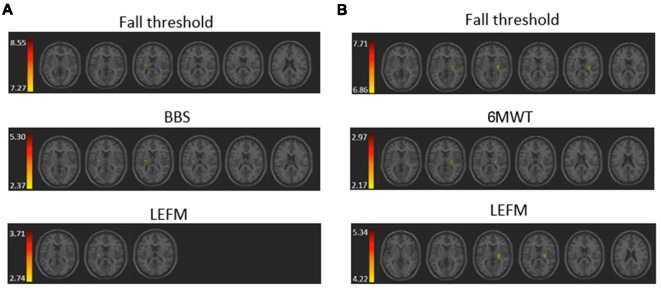
Voxel-based lesion symptom mapping (VLSM) analysis depicting areas where damage was associated with a significant (pFDR <0.05) impact on **(A)** fall threshold, Berg Balance Scale (BBS), and Lower Extremity Fugl-Meyer (LEFM) scores in the RHD group, and **(B)** fall threshold, 6 Minute Walk Test (6MWT) and LEFM scores in the LHD group. Colored regions showed a significant impact on behavioral scores (minimum cluster size: 10 voxels, minimum number of patients: 10). Warmer colors indicate higher z-scores.

The VLSM analysis identified clusters of voxels associated with poorer balance, gait and lower-extremity function (Figure 3). Tables s 3, 4 show the anatomical structures in the right and left hemispheres, respectively, where damage was found to exert a significant impact on the tested functions. In both groups, the major impact is attributed to lesions of the putamen and white matter regions along the corticospinal tract (CST). In the RHD group (Table 3), fall threshold, BBS and LEFM were affected by lesions in the posterior limb of internal capsule (PLIC) and superior corona radiata (SCR), with BBS scores being affected also by damage to the putamen. In the LHD group (Table 4) fall threshold, 6MWT and LEFM were affected by damage to the PLIC and SCR as well as the putamen and EC.

**Table 3:** Voxel-based lesion symptom mapping (VLSM) results in right hemisphere damaged (RHD) patients, *n* = 24.

Test	Structure	*Z*-value	*X*	*Y*	*Z*	Voxels	%area
Fall threshold	PLIC	8.55	26	−14	14	55	10.98
	SCR	7.27	26	−12	20	14	1.52
BBS	PLIC	5.30	26	−14	14	78	15.57
	SCR	3.54	24	−16	20	16	1.74
	Putamen	3.48	28	−14	6	10	0.94
6MWT	VLSM did not yield “significant” voxels						
LEFM	PLIC	3.71	22	−12	14	26	5.19

*Results that passed the FDR correction for multiple comparisons (corresponding to z-scores of 6.440, 2.027 and 2.491 for fall threshold, BBS and LEFM, respectively). Abbreviations: BBS, Berg Balance Scale; 6MWT, 6 Minute Walk Test; LEFM, Lower Extremity Fugl-Meyer Scale; PLIC, posterior limb of internal capsule; SCR, superior corona radiata*.

**Table 4:** Voxel-based lesion symptom mapping (VLSM) results in left hemisphere damaged (LHD) patients, *n* = 22.

Test	Structure	*Z*-value	*X*	*Y*	*Z*	Voxels	%area
Fall threshold	PLIC	7.71	−26	−14	8	73	15.30
	Putamen	7.71	−26	−14	8	38	3.77
	EC	7.71	−30	−12	10	25	5.56
BBS	VLSM did not yield “significant” voxels						
6MWT	PLIC	2.95	−26	−12	12	61	12.79
	Putamen	2.95	−26	−12	12	24	2.38
	EC	2.61	−28	−16	12	22	4.89
	SCR	2.97	−22	−8	24	21	16.54
LEFM	PLIC	5.34	−26	−12	12	108	22.64
	Putamen	5.34	−26	−12	12	58	5.75
	EC	5.22	−28	−12	14	36	8.00
	SCR	4.35	−26	−8	24	23	2.49

*Results that passed the FDR correction for multiple comparisons (corresponding to Z scores of 6.461, 1.908 and 2.571 for fall threshold, 6MWT and LEFM, respectively. Abbreviations: BBS, Berg Balance Scale; 6MWT, 6 Minute Walk Test; LEFM, Lower Extremity Fugl-Meyer Scale; PLIC, posterior limb of internal capsule; EC, external capsule; SCR, superior corona radiata*.

## Discussion

The aim of the current study was to examine the effect of stroke lesions on reactive and anticipatory balance control, gait, and lower limb function. VLSM analysis, conducted separately for patients with right and left hemisphere damage (RHD, LHD), points to a significant impact of damage to the corticospinal tract (in its passage along the corona radiata and/or the posterior limb of the internal capsule) on the tested functions. Thus, in the RHD group, CST damage significantly affected patients’ scores in reactive balance testing (i.e., fall threshold), anticipatory balance control (BBS test results), and hemiparetic lower-limb function (LEFM test results), and in the LHD group it affected patients’ fall threshold, LEFM, and gait velocity (6MWT). Two other subcortical structures where damage was found to affect test results (mainly in the LHD group) are the putamen and EC.

The analysis did not reveal cortical regions containing “significant” voxels (i.e., voxels whose damage makes a significant difference when comparing test results of patients affected and not-affected in these voxels)—for any of the four tested functions (Fall threshold, BBS, 6MWT, LEFM). It should be noted that in VLSM analyses, lack of “significant” voxels within a structure or a brain region does not necessarily indicate that this structure/region does not contribute to the tested function. The need to introduce correction for multiple comparisons, inherent to this mass univariate, voxel-by-voxel analysis, often leads to false-negative results. Thus, the fact that no cortical regions (including the sensory-motor cortex) emerged in the VLSM analysis probably reflects a relatively weaker or inconsistent structure-function relationship that did not survive the FDR correction for multiple comparisons. Yet, the fact that most patients had lesions within the territory of the middle cerebral artery (MCA; sparing the lower-limb representation in the sensory-motor cortex) could also lead to non-demonstration of “significant” cortical voxel clusters.

The apparent similarity of RHD and LHD groups’ lesion patterns (i.e., dominance of MCA territory damage and maximal lesion overlap in the capsular-putaminal region in both groups), as well as the similarity of distribution of “significant” voxel clusters emerging from the VLSM analysis—is incomplete. Thus, VLSM failed to reveal “significant” voxels for the 6MWT in the RHD group and for the BBS in the LHD group. While this is likely to reflect insufficient statistical power, it may still point to hemispheric differences. Also, note that the clusters of “significant” voxels were larger, and generally occupied greater portions of the involved structures, in LHD compared to the RHD patients.

To our knowledge, this is the first study to examine the effect of lesion characteristics on reactive balance control in PwS. The ability to respond effectively to unexpected loss of balance (i.e., balance perturbations) is critical for fall prevention (Maki and McIlroy, 1996). Here, we examined reactive balance capacity by assessing the fall threshold (i.e., fall into the harness system) in response to increased intensities of unannounced surface translations. The results demonstrate that damage in the PLIC and SCR in the RHD group, and in the PLIC, putamen and EC in the LHD group, constitute a negative prognostic factor, being associated with falls into the harness system at lower intensities of surface perturbations.

Balance control was also tested by the BBS, which is a widely used clinical measure of balance control predicting fall risk in stroke patients. The VLSM analysis demonstrated that in RHD patients lower BBS scores are related to lesions involving the PLIC, SCR and the putamen. In contra-distinction, VLSM failed to disclose “significant” voxels in the LHD group, a finding that possibly points to differences in the neural mechanisms taking place in balance control in the non-dominant and dominant hemispheres, with a larger underlying structural basis (and substitution options) in the latter. The difference in distribution of “significant” voxels affecting fall threshold vs. BBS test results is likely to indicate that reactive and anticipatory balance control do not share exactly the same neural mechanisms. Reactive balance control was assessed here by measuring the fall threshold in response to surface translations that were unpredicted in terms of timing and direction. In contrast, the BBS assesses balance in conditions where timing and direction of center of mass displacement are known in advance, prior to movement initiation, thus recruiting a more anticipatory mode of control (though, certainly maintaining a reactive component in it due to the fact that predictions are not fully accurate and online corrections based on afferent input are needed). In a previous VLSM research by Reynolds et al. (2014), lower BBS test results were associated with damage to the caudate, putamen, pallidum and thalamus as well as to cortical damage in the precentral gyrus, frontal operculum and the cuneus. However, in that study RHD brains were flipped to the left hemisphere (seemingly assuming equal hemispheric contribution to balance control) and the total sample size was relatively small (*n* = 16). In another recent study by Moon et al. (2016) conducted in a group of subacute stroke patients (as in the current study), the analysis failed to disclose a significant impact of damage to a given brain structure on BBS test results, despite examination of a large cohort (*n* = 133).

Upright stability is considered to be controlled by a broad and distributed neural network. However, as shown by discrepancies in the results of the above lesion studies, the relative contribution of distinct parts of the nervous system to maintenance of the upright posture remains unclear (Bolton, 2015). Our results demonstrate the importance of subcortical regions—mainly the corticospinal tract, and to a lesser extent the putamen and EC, in balance control. Diffuse periventricular white matter changes (often involving the corona radiata) were found in past research to be a strong risk factor for falls in the general older population (Srikanth et al., 2009) and were associated with impaired balance as measured with the single-leg balance test (Starr et al., 2003). Previous studies suggested that the basal ganglia, in their role as mediators of motor-planning processes, act as an intermediary between the cerebral cortex and the brainstem descending pathways for automating the selection and execution of an optimal reactive balance response (Takakusaki et al., 2004; Grillner et al., 2005; Jacobs and Horak, 2007).

The neural substrate that supports balance control was also found to be important for gait velocity (6MWT results) and lower limb function (LEFM), as the latter two were also affected by damage to voxel clusters involving the corticospinal tract as well as the putamen and EC. However, this pattern was revealed in the LHD group, whereas in the RHD group LEFM scores were affected by damage restricted to the posterior limb of the internal capsule, and no voxels were found to relate significantly to 6MWT results. Our results are in line with a previous study (Jones et al., 2016) demonstrating that poor recovery of gait speed was predicted by lesions involving the insula, putamen, EC, the superior longitudinal fasciculus, inferior fronto-occipital fasciculus and uncinate fasciculus. In another study, gait speed was found to be affected by damage to the insula, the internal capsule and to the adjacent white matter (Moon et al., 2017). In addition to gait speed, few studies used the FAC to assess mobility. Lee et al. (2017) demonstrated that damage to the corona radiata, internal capsule, globus pallidus, putamen and cingulum was related to poor recovery as indicated by a lower score in the FAC, at 3 months after stroke onset. Also, damage to the putamen, insula and EC was related to temporal gait asymmetry (Alexander et al., 2009).

The current VLSM analysis revealed, as expected, a significant negative impact of CST damage on the function of the hemiparetic lower limb (LEFM score). The “significant” voxel clusters were located in the posterior limb of the internal capsule (PLIC), both in RHD and LHD patient groups. In the latter group, LEFM scores were affected, in addition, by damage to voxel clusters within the EC, putamen, and superior corona radiate. In earlier research, lower extremity motor impairment was reported to relate to CST lesions either in the PLIC or in the corona radiata (Lee et al., 2005; Alexander et al., 2009; Jayaram et al., 2012). Moon et al. (2016) showed that lower LEFM scores were associated with damage to the lentiform and caudate nuclei, insula, internal capsule and corona radiate in both hemispheres (much in common with the findings of the LHD group in the current study).

Several limitations of the study should be acknowledged. First, the results are based on a moderate sample size (*n* = 46) and are restricted by the inclusion criteria of the study to stroke of mild to moderate severity. Second, PwS were included in the study as soon as they were able to stand and walk independently, or under supervision, and consequently, time-after-onset was not constant (yet, within the subacute phase). Assessment of the effect of lesion characteristics in the chronic stage will necessitate a separate study. It should be noted that the large majority of patients in the current cohort had strokes located within the MCA territory, thus restricting the possibility to identify “significant” voxel clusters largely to that territory. Analysis of a cohort including sufficient patients damaged in the territory of the anterior cerebral artery (ACA) is most likely to reveal a significant impact of damage to portions of the sensory-motor cortex where the cell bodies of the CST neurons controlling lower-limb movement reside. Similarly, analysis of a cohort of stroke patients damaged in the brain stem is likely to reveal the importance of structures and descending pathways that could not emerge in the analysis of the current patient groups.

In conclusion, the study corroborates and extends previous findings by demonstrating that impairments in balance control, gait velocity, and hemiparetic lower limb motor performance are associated with lesions to the CST in its passage through the posterior limb of the internal capsule and the corona radiate. Other structures within the MCA territory, where damage exerts a significant impact on the tested functions, are the putamen and EC. The question of differential lesion effects in the dominant and non-dominant hemispheres need further assessment.

## Ethics Statement

The study was approved by the Loewenstein Rehabilitation Hospital Review Board (#LOE-14-0021).

## Author Contributions

SH was involved in planning and conducting the experiments as well as data analysis, interpretation and drafting of the manuscript. IM was involved in planning the experiments and drafting of the manuscript. NS was involved in subject medical screening, lesion delineation as well as data analysis, interpretation and drafting of the manuscript. All authors read and approved the final manuscript.

## Conflict of Interest Statement

IM owns a patent on some of the technology used in the BalanceTutor system and receives a part of the standard royalty distribution for the BalanceTutor system. The remaining authors declare that the research was conducted in the absence of any commercial or financial relationships that could be construed as a potential conflict of interest.

## References

[B1] Alexander L. D. Black S. E. Patterson K. K. Gao F. Danells C. J. McIlroy W. E. (2009). Association between gait asymmetry and brain lesion location in stroke patients. Stroke 40, 537–544. 10.1161/STROKEAHA.108.527374 19109546

[B2] Batchelor F. A. Mackintosh S. F. Said C. M. Hill K. D. (2012). Falls after stroke. Int. J. Stroke 7, 482–490. 10.1111/j.1747-4949.2012.00796.x 22494388

[B3] Bates E. Wilson S. M. Saygin A. P. Dick F. Sereno M. I. Knight R. T.. (2003). Voxel-based lesion-symptom mapping. Nat. Neurosci. 6, 448–450. 10.1038/nn1050 12704393

[B4] Belgen B. Beninato M. Sullivan P. E. Narielwalla K. (2006). The association of balance capacity and falls self-efficacy with history of falling in community-dwelling people with chronic stroke. Arch. Phys. Med. Rehabil. 87, 554–561. 10.1016/j.apmr.2005.12.027 16571397

[B5] Benjamini Y. Hochberg Y. (1995). Controlling the false discovery rate: a practical and powerful approach to multiple testing. J. R. Stat. Soc. B 57, 289–300. 10.1111/j.2517-6161.1995.tb02031.x

[B6] Berg K. Wood-Dauphinee S. Williams J. I. (1995). The balance scale: reliability assessment with elderly residents and patients with an acute stroke. Scand. J. Rehabil. Med. 27, 27–36. 7792547

[B7] Bolton D. A. (2015). The role of the cerebral cortex in postural responses to externally induced perturbations. Neurosci. Biobehav. Rev. 57, 142–155. 10.1016/j.neubiorev.2015.08.014 26321589

[B8] Butland R. J. Pang J. Gross E. R. Woodcock A. A. Geddes D. M. (1982). Two-, six- and 12-minute walking tests in respiratory disease. Br. Med. J. 284, 1607–1608. 10.1136/bmj.284.6329.1607 6805625 PMC1498516

[B9] Cheng B. Forkert N. D. Zavaglia M. Hilgetag C. C. Golsari A. Siemonsen S.. (2014). Influence of stroke infarct location on functional outcome measured by the modified ranking scale. Stroke 45, 1695–1702. 10.1161/STROKEAHA.114.005152 24781084

[B10] Davenport R. J. Dennis M. S. Wellwood I. Warlow C. P. (1996). Complications after acute stroke. Stroke 27, 415–420. 10.1161/01.STR.27.3.415 8610305

[B11] de Kam D. Roelofs J. M. B. Bruijnes A. K. B. D. Geurts A. C. H. Weerdesteyn V. (2017). The next step in understanding impaired reactive balance control in people with stroke: the role of defective early automatic postural responses. Neurorehabil. Neural Repair 31, 708–716. 10.1177/1545968317718267 28691582 PMC5714159

[B12] Duncan P. W. Goldstein L. B. Matchar D. Divine G. W. Feussner J. (1992). Measurement of motor recovery after stroke. Outcome assessment and sample size requirements. Stroke 23, 1084–1089. 10.1161/01.str.23.8.1084 1636182

[B13] Eng J. J. Dawson A. S. Chu K. S. (2004). Submaximal exercise in persons with stroke: test-retest reliability and concurrent validity with maximal oxygen consumption. Arch. Phys. Med. Rehabil. 85, 113–118. 10.1016/s0003-9993(03)00436-2 14970978 PMC3167868

[B14] Flansbjer U. B. Blom J. Brogardh C. (2012). The reproducibility of berg balance scale and the single-leg stance in chronic stroke and the relationship between the two tests. PM R 4, 165–170. 10.1016/j.pmrj.2011.11.004 22306324

[B15] Flansbjer U. B. Holmbäck A. M. Downham D. Patten C. Lexell J. (2005). Reliability of gait performance tests in men and women with hemiparesis after stroke. J. Rehabil. Med. 37, 75–82. 10.1080/16501970410017215 15788341

[B16] Forster A. Young J. (1995). Incidence and consequences of falls due to stroke: a systematic inquiry. BMJ 311, 83–86. 10.1136/bmj.311.6997.83 7613406 PMC2550147

[B17] Fugl-Meyer A. R. Jääskö L. Leyman I. Olsson S. Steglind S. (1975). The post-stroke hemiplegic patient: a method for evaluation of physical performance. Scand. J. Rehabil. Med. 7, 13–31. 1135616

[B18] Grillner S. Hellgren J. Ménard A. Saitoh K. Wikström M. A. (2005). Mechanisms for selection of basic motor programs-roles for the striatum and pallidum. Trends Neurosci. 28, 364–370. 10.1016/j.tins.2005.05.004 15935487

[B19] Haramati S. Soroker N. Dudai Y. Levy D. A. (2008). The posterior parietal cortex in recognition memory: a neuropsychological study. Neuropsychologia 46, 1756–1766. 10.1016/j.neuropsychologia.2007.11.015 18178228

[B20] Honeycutt C. F. Nevisipour M. Grabiner M. D. (2016). Characteristics and adaptive strategies linked with falls in stroke survivors from analysis of laboratory-induced falls. J. Biomech. 49, 3313–3319. 10.1016/j.jbiomech.2016.08.019 27614614 PMC5074874

[B21] Inness E. L. Mansfield A. Lakhani B. Bayley M. McIlroy W. E. (2014). Impaired reactive stepping among patient ready for discharge from inpatient stroke rehabilitation. Phys. Ther. 94, 1755–1764. 10.2522/ptj.20130603 25104795 PMC4263904

[B22] Jacobs J. V. Horak F. B. (2007). Cortical control of postural responses. J. Neural. Transm. 114, 1339–1348. 10.1007/s00702-007-0657-0 17393068 PMC4382099

[B23] Jayaram G. Stagg C. J. Esser P. Kischka U. Stinear J. Johansen-Berg H. (2012). Relationships between functional and structural corticospinal tract integrity and walking post stroke. Clin. Neurophysiol. 123, 2422–2428. 10.1016/j.clinph.2012.04.026 22717679 PMC3778984

[B24] Jones P. S. Pomeroy V. M. Wang J. Schlaug G. Tulasi Marrapu S. Geva S.. (2016). Does stroke location predict walk speed response to gait rehabilitation? Hum. Brain Mapp. 37, 689–703. 10.1002/hbm.23059 26621010 PMC4738376

[B25] Lancaster J. L. Woldorff M. G. Parsons L. M. Liotti M. Freitas C. S. Rainey L.. (2000). Automated talairach atlas labels for functional brain mapping. Hum. Brain Mapp. 10, 120–131. 10.1002/1097-0193(200007)10:3<120::AID-HBM30>3.0.CO;2-8 10912591 PMC6871915

[B26] Langhorne P. Stott D. J. Robertson L. MacDonald J. Jones L. McAlpine C.. (2000). Medical complications after stroke: a multicenter study. Stroke 31, 1223–1229. 10.1161/01.str.31.6.1223 10835436

[B27] Lee J. S. Han M. K. Kim S. H. Kwon O. K. Kim J. H. (2005). Fiber tracking by diffusion tensor imaging in corticospinal tract stroke: topographical correlation with clinical symptoms. Neuroimage 26, 771–776. 10.1016/j.neuroimage.2005.02.036 15955486

[B28] Lee K. B. Kim J. S. Hong B. Y. Sul B. Song S. Sung W. J.. (2017). Brain lesions affecting gait recovery in stroke patients. Brain Behav. 7:e00868. 10.1002/brb3.868 29201557 PMC5698874

[B29] Lo R. Gitelman D. Levy R. Hulvershorn J. Parrish T. (2010). Identification of critical areas for motor function recovery in chronic stroke subjects using voxel-based lesion symptom mapping. Neuroimage 49, 9–18. 10.1016/j.neuroimage.2009.08.044 19716427

[B30] Maki B. E. McIlroy W. E. (1996). Postural control in the older adult. Clin. Geriatr. Med. 12, 635–658. 10.1016/s0749-0690(18)30193-9 8890108

[B31] Maki B. E. McIlroy W. E. (1997). The role of limb movements in maintaining upright stance: the “change-in-support” strategy. Phys. Ther. 77, 488–507. 10.1093/ptj/77.5.488 9149760

[B32] Maki B. E. McIlroy W. E. (2006). Control of rapid limb movements for balance recovery: age-related changes and implications for fall prevention. Age Ageing 35, ii12–ii18. 10.1093/ageing/afl078 16926197

[B33] Mansfield A. Inness E. L. Wong J. S. Fraser J. E. McIlroy W. E. (2013). Is impaired control of reactive stepping related to falls during inpatient stroke rehabilitation? Neurorehabil. Neural Repair 27, 526–533. 10.1177/1545968313478486 23504551

[B34] Marigold D. S. Eng J. J. (2006). Altered timing of postural reflexes contributes to falling in persons with chronic stroke. Exp. Brain Res. 171, 459–468. 10.1007/s00221-005-0293-6 16418855 PMC3226801

[B35] Martinez K. M. Mille M. L. Zhang Y. Rogers M. W. (2013). Stepping in persons poststroke: comparison of voluntary and perturbation-induced responses. Arch. Phys. Med. Rehabil. 94, 2425–2432. 10.1016/j.apmr.2013.06.030 23872077

[B36] McDonald V. Hauner K. K. Chau A. Krueger F. Grafman J. (2017). Networks underlying trait impulsivity: evidence from voxel-based lesion-symptom mapping. Hum. Brain Mapp. 38, 656–665. 10.1002/hbm.23406 27667777 PMC5225118

[B37] Medina J. Kimberg D. Y. Chatterjee A. Coslett H. B. (2010). Inappropriate usage of the Brunner-Munzel test in recent voxel-based lesion-symptom mapping studies. Neuropsychologia 48, 341–343. 10.1016/j.neuropsychologia.2009.09.016 19766664 PMC2795086

[B38] Meyer S. Kessner S. S. Cheng B. Bönstrup M. Schulz R. Hummel F. C.. (2015). Voxel-based lesion-symptom mapping of stroke lesions underlying somatosensory deficits. Neuroimage Clin. 10, 257–266. 10.1016/j.nicl.2015.12.005 26900565 PMC4724038

[B39] Michael K. M. Allen J. K. Macko R. F. (2005). Reduced ambulatory activity after stroke: the role of balance, gait and cardiovascular fitness. Arch. Phys. Med. Rehabil. 86, 1552–1556. 10.1016/j.apmr.2004.12.026 16084807

[B40] Moon H. I. Lee H. J. Yoon S. Y. (2017). Lesion location associated with balance recovery and gait velocity change after rehabilitation in stroke patients. Neuroradiology 59, 609–618. 10.1007/s00234-017-1840-0 28523357

[B41] Moon H. I. Pyun S. B. Tae W. S. Kwon H. K. (2016). Neural substrates of lower extremity motor, balance and gait function after supratentorial stroke using voxel-based lesion symptom mapping. Neuroradiology 58, 723–731. 10.1007/s00234-016-1672-3 26961307

[B42] Pouwels S. Lalmohamed A. Leufkens B. de Boer A. Cooper C. van Staa T.. (2009). Risk of hip/femur fracture after stroke: a population based case-control study. Stroke 40, 3281–3285. 10.1161/STROKEAHA.109.554055 19661475

[B43] Reynolds A. M. Peters D. M. Vendemia J. M. Smith L. P. Sweet R. C. Baylis G. C.. (2014). Neuronal injury in the motor cortex after chronic stroke and lower limb motor impairment: a voxel-based lesion symptom mapping study. Neural Regen. Res. 9, 766–772. 10.4103/1673-5374.131589 25206888 PMC4146271

[B44] Rorden C. Karnath H. O. Bonilha L. (2007). Improving lesion-symptom mapping. J. Cogn. Neurosci. 19, 1081–1088. 10.1162/jocn.2007.19.7.1081 17583985

[B45] Salot P. Patel P. Bhatt T. (2015). Reactive balance in individuals with chronic stroke: biomechanical factors related to perturbation-induced backward falling. Phys. Ther. 96, 338–347. 10.2522/ptj.20150197 26206220

[B46] Simpson L. A. Miller W. C. Eng J. J. (2011). Effect of stroke on fall rate, location and predictors: prospective comparison of older adults with and without stroke. PLoS One 6:e19431. 10.1371/journal.pone.0019431 21559367 PMC3084849

[B47] Solomon J. Raymont V. Braun A. Butman J. A. Grafmanc J. (2007). User-friendly software for the analysis of brain lesions (ABLe). Comput. Methods Programs Biomed. 86, 245–254. 10.1016/j.cmpb.2007.02.006 17408802 PMC1995425

[B48] Srikanth V. Beare R. Blizzard L. Phan T. Stapleton J. Chen J.. (2009). Cerebral white matter lesions, gait and the risk of incident falls: a prospective population-based study. Stroke 40, 175–180. 10.1161/STROKEAHA.108.524355 18927448

[B49] Starr J. M. Leaper S. A. Murray A. D. Lemmon H. A. Staff R. T. Deary I. J.. (2003). Brain white matter lesions detected by magnetic resosnance imaging are associated with balance and gait speed. J. Neurol. Neurosurg. Psychiatry 74, 94–98. 10.1136/jnnp.74.1.94 12486275 PMC1738198

[B50] Takakusaki K. Oohinata-Sugimoto J. Saitoh K. Habaguchi T. (2004). Role of basal ganglia-brainstem systems in the control of postural muscle tone and locomotion. Prog. Brain Res. 143, 231–237. 10.1016/s0079-6123(03)43023-9 14653168

[B51] Tzourio-Mazoyer N. Landeau B. Papathanassiou D. Crivello F. Etard O. Delcroix N.. (2002). Automated anatomical labeling of activations in SPM using a macroscopic anatomical parcellation of the MNI MRI single-subject brain. Neuroimage 15, 273–289. 10.1006/nimg.2001.0978 11771995

[B52] van Duijnhoven H. J. Heeren A. Peters M. A. Veerbeek J. M. Kwakkel G. Geurts A. C.. (2016). Effects of exercise therapy on balance capacity in chronic stroke: systematic review and meta-analysis. Stroke 47, 2603–2610. 10.1161/STROKEAHA.116.013839 27633021

[B53] Weerdesteyn V. de Niet M. van Duijnhoven H. J. Geurts A. C. (2008). Falls in individuals with stroke. J. Rehabil. Res. Dev. 45, 1195–1213. 10.1682/JRRD.2007.09.0145 19235120

[B54] Winstein C. J. Stein J. Arena R. Bates B. Cherney L. R. Cramer S. C. (2016). Guidelines for adult stroke rehabilitation and recovery: a guideline for healthcare professionals from the American Heart Association/American Stroke Association. Stroke 47, e98–e169. 10.1161/STR.0000000000000098 27145936

[B55] Xu T. Clemson L. O’Loughlin K. Lannin N. A. Dean C. Koh G. (2018). Risk factors for falls in community stroke survivors: a systematic review and meta-analysis. Arch. Phys. Med. Rehabil. 99, 563–573. 10.1016/j.apmr.2017.06.032 28797618

[B56] Zhu L. L. Lindenberg R. Alexander M. P. Schlaug G. (2010). Lesion load of the corticospinal tract predicts motor impairment in chronic stroke. Stroke 41, 910–915. 10.1161/STROKEAHA.109.577023 20378864 PMC2886713

