# Supplementary material for: Analysis of Brain Lesion Impact on Balance and Gait Following Stroke
Source: Front Hum Neurosci. 2019 May 14;13:149. doi: 10.3389/fnhum.2019.00149 (PMC6527742; doi:10.3389/fnhum.2019.00149)
Supplement: Supplementary file 1 [file Data_Sheet_1.docx]

Supplementary Material

**Analysis of Brain Lesion Impact on Balance and Gait Following Stroke**

Shirley Handelzalts^*^, Itshak Melzer, Nachum Soroker

***Correspondence:** Shirley Handelzalts: peregshir@gmail.com

1. Supplementary Figure
2. Tables 1a and 1b.


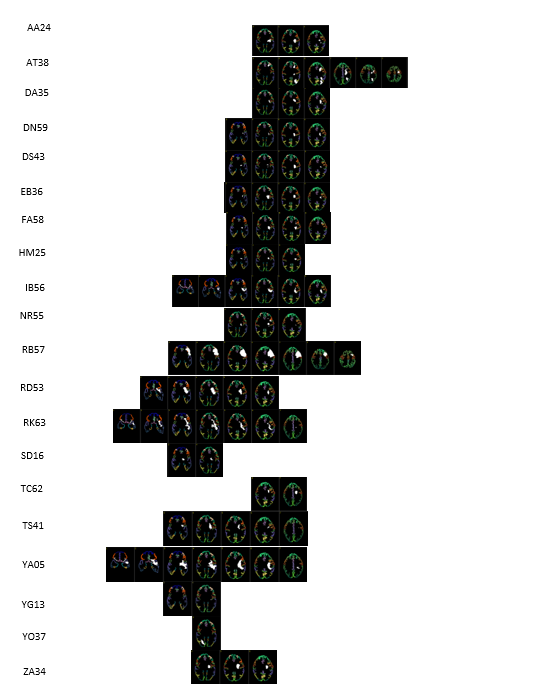

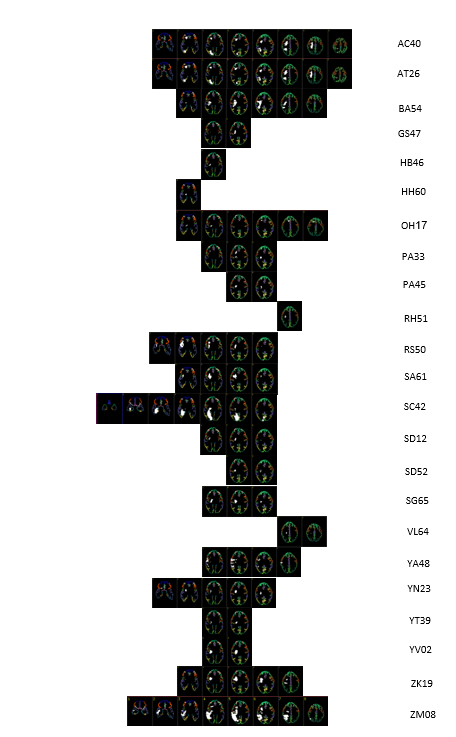


**Supplementary Figure 1**. Each patient's lesion marked on arrays of 11 standard templates. Displays follow neurological conventions, i.e., right sided damage displayed on the left and left sided damage displayed on the right side. Only CT slices that present brain damage are shown. Three cases with damage restricted to posterior-fossa structures above the pyramidal decussation, are not shown (patient MN66 with right sided damage, lesion size 4.39cc; and patients MO49 and IK11 with left-sided damage, lesion size 10.32cc and 2.62cc, respectively).

| **LHD group (n=22)** | | **RHD group (n=24)** | | **Structure** |
| --- | --- | --- | --- | --- |
| **Median (range)** | **n** | **Median (range)** | **n** |  |
| 0.26(0.06-55.2) | 5 | 0.98(0.2-3.1) | 5 | Precentral |
| 2.87(2.7-3.1) | 2 | 0.41(0.1-3.4) | 4 | Frontal superior |
| 5.09 | 1 | - | 0 | Frontal superior orbital |
| 3.56(0.3-36.8) | 3 | 0.33(0.04-0.45) | 7 | Frontal middle |
| - | 0 | 0.1 | 1 | Frontal middle orbital |
| 2.90(2.2-95.6) | 5 | 4.93(0.3-19.3) | 7 | Frontal inferior opercularis |
| 0.16(0.1-79.7) | 6 | 2.25(1.8-7.5) | 4 | Frontal inferior triangular |
| 0.83(0.5-1.5) | 3 | 0.32(0.06-0.6) | 2 | Frontal_Inferior orbital |
| 1.92(0.1-42.6) | 8 | 2.55(0.08-28.4) | 9 | Rolandic operculum |
| - | 0 | 2.88(0.08-5.7) | 2 | Supplementary motor area |
| 5.0(1.8-8.2) | 2 | 0.69 | 1 | Olfactory |
| 0.07 | 1 | 0.93(0.14-1.73) | 2 | Frontal superior medial |
| - | 0 | 0.12 | 1 | Frontal middle_orbital |
| 6.81 | 1 | - | 0 | Rectus |
| 7.0(0.81-53.2) | 12 | 3.16(0.06-38.6) | 16 | Insula |
| - | 0 | 0.87(0.3-1.4) | 2 | Cingulum anterior |
| 0.46(0.05-1.1) | 3 | 0.63(0.4-2.3) | 4 | Cingulum middle |
| - | 0 | - | 0 | Cingulum posterior |
| 0.48(0.2-31.5) | 6 | 8.03(0.5-64.2) | 3 | Hippocampus |
| 0.31 | 1 | 21.3(0.2-42.5) | 2 | ParaHippocampal |
| 15.4(2.7-43.2) | 3 | 1.21(0.8-1.6) | 2 | Amygdala |
| 2.17 | 1 | 4.41(0.5-30.9) | 3 | Calcarine |
| 0.07 | 1 | 0.35(0.3-1.4) | 3 | Cuneus |
| 0.62 | 1 | 11.96(3.3-65.5) | 3 | Lingual |
| 3.2 | 1 | 0.99(0.2-1.0) | 3 | Occipital superior |
| 10.2(0.03-20.5) | 2 | 0.10(0.1-20.7) | 3 | Occipital middle |
| 12.33 | 1 | 11.8(11.4-26.9) | 3 | Occipital inferior |
| 0.13(0.09-0.7) | 3 | 19.26(5.2-75.8) | 3 | Fusiform |
| 8.9(0.5-17.2) | 2 | 0.68(0.08-5.7) | 5 | Postcentral |
| - | 0 | 0.14 | 1 | Parietal superior |
| - | 0 | 0.59 | 1 | Parietal inferior |
| 0.16(0.08-2.1) | 3 | 1.67(0.5-6.3) | 5 | SupraMarginal |
| 0.26(0.09-0.4) | 2 | 8.3(5.2-18.8) | 3 | Angular |
| 0.09 | 1 | 0.46(0.4-0.5) | 3 | Precuneus |
| 0.15 | 1 | - | 0 | Paracentral lobule |
| 2.70(0.1-29.1) | 15 | 2.16(0.4-10.6) | 14 | Caudate |
| 15.4(0.1-95.9) | 16 | 9.21(0.09-52.6) | 17 | Putamen |
| 11.26(0.7-67.6) | 14 | 3.39(0.4-39.6) | 14 | Pallidum |
| 7.18(0.09-31.9) | 16 | 1.80(0.09-18.2) | 17 | Thalamus |
| 15.55(0.9-42.2) | 8 | 8.84(2.0-14.1) | 5 | Heschl |
| 3.70(0.6-16.4) | 7 | 2.53(0.03-55.4) | 6 | Temporal superior |
| 1.87(0.6-4.8) | 5 | 0.97(0.9-2.1) | 3 | Temporal pole superior |
| 2.79(0.2-17.4) | 5 | 5.04(1.9-63.0) | 4 | Temporal middle |
| 3.57(0.2-6.9) | 2 | 1.94 | 1 | Temporal pole middle |
| 6.39(3.0-9.7) | 2 | 3.79(0.2-25.7) | 4 | Temporal inferior |
| 1.97(0.1-3.8) | 2 | 2.87(1.2-7.0) | 3 | Cerebelum crus 1 |
| 26.53(15.8-37.2) | 2 | 3.31 | 1 | Cerebelum crus 2 |
| 1.47 | 1 | 0.97 | 1 | Cerebelum 3 |
| - | 0 | 12.08 | 1 | Cerebelum 4 5 |
| - | 0 | 9.3(2.4-10.2) | 3 | Cerebelum 6 |
| 44.02(36.1-52.0) | 2 | - | 0 | Cerebelum 7b |
| 53.07(51.4-54.7) | 2 | 11.96 | 1 | Cerebelum 8 |
| 40.45(31.4-49.5) | 2 | 2.22 | 1 | Cerebelum 9 |
|  | 0 | - | 0 | Cerebelum 10 |
| 1.15(1.15-1.15) | 2 | - | 0 | Vermis |

**Table 1a**. Percentage of structure impacted by lesion in right hemisphere damaged (RHD) and left hemisphere damaged (LHD) patients.

Reports from the Automated Anatomical Labeling (AAL) atlas

No significant differences were found between groups (Mann-Whitney U test, Bonferroni correction for multiple comparisons).

**Table 1b**. Percentage of structure impacted by lesion in right hemisphere damaged (RHD) and left hemisphere damaged (LHD) patients.

| **LHD group (n=22)** | | **RHD group (n=24)** | | **Structure** |
| --- | --- | --- | --- | --- |
| **Median (range)** | **n** | **Median (range)** | **n** |  |
| 37.64 | 1 | - | 0 | Corticospinal tract |
| 26.51 | 1 | - | 0 | Medial leminiscus |
| 1.68 | 1 | - | 0 | Inferior cerebellar peduncle |
| 7.44 | 1 | - | 0 | Superior cerebellar peduncle |
| 6.08(0.4-42.6) | 9 | 9.33(1.5-12.3) | 6 | Cerebral peduncle |
| 14.28(6.4-83.2) | 8 | 4.54(0.2-42.7) | 14 | Anterior limb of internal capsule |
| 38.57(0.21-79.9) | 17 | 23.6(4.8-60.5) | 18 | Posterior limb of internal capsule |
| 18.97(0.3-97.7) | 15 | 7.28(0.3-85.1) | 11 | Retrolenticular part of internal capsule |
| 3.75(0.2-33.2) | 8 | 11.10(1.9-60.9) | 8 | Anterior corona radiate |
| 22.8(0.9-47.1) | 15 | 12.07(0.2-47.7) | 17 | Superior corona radiate |
| 8.07(1.3-46.9) | 10 | 7.74(1.5-27.6) | 11 | Posterior corona radiate |
| 31.69(30.5-32.8) | 2 | 21.7(0.6-66.5) | 5 | Posterior thalamic radiation |
| 20.6(2.1-62.5) | 4 | 20.63(1.0-83.6) | 4 | Sagittal stratum |
| 25.0(0.44-90.0) | 16 | 13.62(2.4-58.4) | 16 | External capsule |
| - | 0 | 50.3 | 1 | Cingulum (in the hippocampal region) |
| 3.40(0.7-84.3) | 8 | 2.19(0.7-69.3) | 5 | Fornix and stria terminalis |
| 2.58(0.7-49.1) | 13 | 9.70(0.2-72.7) | 13 | Superior longitudinal fasciculus |
| 34.55(1.8-69.1) | 12 | 22.03(1.7-72.9) | 11 | Superior fronto-occipital fasciculus |
| 23.14(2.1-92.1) | 9 | 13.49(0.4-25.5) | 8 | Inferior fronto-occipital fasciculus |
| 58.16(6.1-97.9) | 6 | 9.57(8.5-10.6) | 2 | Uncinate fasciculus |
| 36.62(21.1-52.1) | 2 | 5.12(2.6-7.7) | 2 | Tapetum |
| 0.69(0.4-5.4) | 6 | 1.16(0.1-1.2) | 5 | Body of corpus callosum |
| 0.37 | 1 | 11.01 | 1 | Middle cerebellar peduncle |
| 0.88(0.2-2.3) | 4 | 1.59(1.1-3.1) | 4 | Genu of the corpus callosum |
| 1.55(0.6-2.5) | 2 | 0.39(0.3-0.4) | 3 | Splenium of the corpus callosum |

Reports from the White Matter atlas.

No significant differences were found between groups (Mann-Whitney U test, Bonferroni correction for multiple comparisons).
